# Morphological and molecular differentiation between *Culicoides oxystoma* and *Culicoides kingi* (Diptera: Ceratopogonidae) in Tunisia

**DOI:** 10.1186/s13071-021-05084-8

**Published:** 2021-12-18

**Authors:** Darine Slama, Rihab Baraket, Latifa Remadi, Emna Chaker, Hamouda Babba

**Affiliations:** Laboratory of Medical and Molecular Parasitology-Mycology LP3M (Code LR12ES08), Department of Clinical Biology B, Faculty of Pharmacy, University of Monastir, 5000 Monastir, Tunisia

**Keywords:** *Culicoides oxystoma*, *Culicoides kingi*, Morphological, Morphometric, Molecular identification, PCR–RFLP, Tunisia

## Abstract

**Background:**

*Culicoides kingi* and *Culicoides oxystoma* belong to the *Schultzei* group of biting midges. These two species are vectors of disease in livestock of economic importance. As described in the literature, morphological identification for discrimination between them is still unclear. However, species-specific identification is necessary to solve taxonomic challenges between species and to understand their roles in disease transmission and epidemiology. This study aims to develop accurate tools to discriminate *C. oxystoma* from *C. kingi* using traditional morphometry and polymerase chain reaction-restriction fragment length polymorphism (PCR RFLP) assays for use in developing countries.

**Methods:**

Specimens were collected from the region of Kairouan in central Tunisia. A total of 446 *C. oxystoma*/*C. kingi* individuals were identified using traditional morphometric analyses combined with PCR–RFLP of the cytochrome c oxidase subunit I gene. Thirteen morphometric measurements were performed from the head, wings, and abdomen of slide-mounted specimens, and six ratios were calculated between these measurements. Multivariate analyses of the morphometric measurements were explored to identify which variables could lead to accurate species identification.

**Results:**

Four variables, namely antennae, wings, spermathecae, and palpus length, were suitable morphometric characteristics to differentiate between the species. Digestion with the SspI restriction enzyme of the PCR product led to good discriminative ability. Molecular procedures and phylogenetic analysis confirmed the efficiency of this simple and rapid PCR–RFLP method.

**Conclusions:**

This study highlights for the first time in Tunisia the presence of *C. oxystoma* and its discrimination from *C. kingi* using abdominal measurements and the PCR–RFLP method. This approach could be applied in future epidemiological studies at the national and international levels.

**Graphical Abstract:**

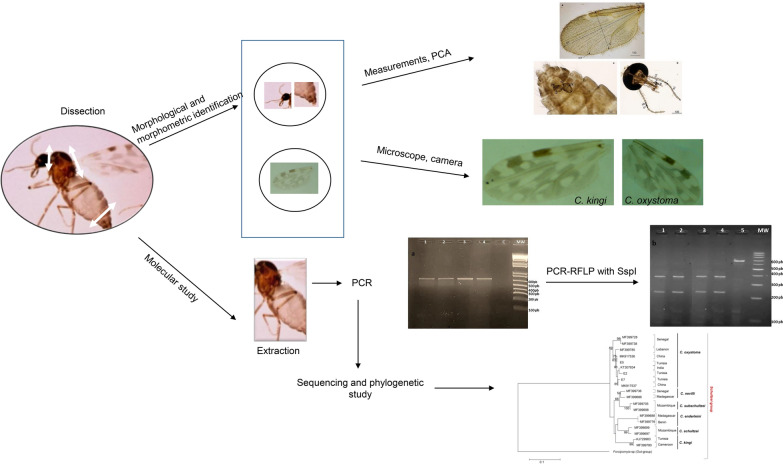

**Supplementary Information:**

The online version contains supplementary material available at 10.1186/s13071-021-05084-8.

## Background

The biting midges in the genus *Culicoides* (Diptera: Ceratopogonidae) are small hematophagous flies (from 1 to 5 mm) that are widely distributed worldwide [1]. This genus includes 1347 valid species. Of these, 875 species are placed in subgenera, 336 species are placed in separate species groups, and 136 species are still unclassified [2].

They are biological vectors of a wide variety of important arbovirus diseases, including bluetongue virus (BTV), African horse sickness (AHS), epizootic hemorrhagic disease virus (EHDV), and Schmallenberg virus (SBV) [3, 4]. These diseases are considered a great threat to livestock, with important economic implications. In both the Mediterranean Basin and sub-Saharan Africa, the main vector of BTV and SBV is *Culicoides imicola* [1]. Other *Culicoides* species, such as *C. obsoletus* and *C. scoticus*, are reportedly able to transmit BTV in regions where *C. imicola* is absent. Moreover, several species of the subgenus *Culicoides*, including *C. pulicaris*, *C. lupicaris*, *C. punctatus*, *C. newsteadi*, and *C. paolae*, are considered potential vectors of BTV based on their ecological habitats and on virus isolation or viral genome detection from field-collected individuals [5].

*Culicoides* groupings such as the *Schultzei* species group (*C. oxystoma*, *C. schultzei*, *C. subschultzei*, *C. kingi*, *C. rhizophorensis*, *C. enderleini*, *C. nevilli*, and *C. neoschultzei*) are implicated in the transmission of some of these viruses [6]. In particular, in Australian and Asian regions, *C. oxystoma* is a vector of bovine arboviruses such as Akabane virus in Japan [7] and EHDV in Israel [8], and is a potential vector of BTV in India [9]. The emergence of these different diseases highlights the need to understand the epidemiology and transmission of *Culicoides*-borne viruses in Tunisia. This understanding requires detailed knowledge of the vector species, including their life characteristics, abundance, and vector competence [10].

In the investigation of vector-borne diseases, the proper identification of arthropod species is very important, and it is critical to differentiate them from other *Culicoides* species. Morphological identification includes many important morphological characteristics, such as the pigmentation pattern of the wings, length and shape of the antennal segments, characteristics of the genitalia in males, distribution of the sensilla on the antennae, and number and size of the spermathecae in females [11, 12]. Specific diagnosis between closely related species is often difficult, requiring intense work from expert taxonomists according to morphological parameters [12], without error [13]. Moreover, damage to midges during the collection step can result in identification difficulties or misidentification.

Consequently, other techniques such as morphometric discrimination [14, 15] and landmark-based geometric morphometrics [16–18] have been developed to separate intractable species. Many studies have used these methods to classify species and to examine variation among medically important mosquitoes that are morphologically similar, such as *Anopheles* spp. [19]. The landmark-based approach is considered the main method for wing venation patterns, helping to improve morphological species identification such as species of the *Maculatus* group [19].

Various biting midge species are found in the same habitat, and they share certain morphological features. *Culicoides oxystoma* shares many morphological features with *C. kingi*, leading to a great deal of confusion. Only expert entomologists can resolve such differentiation, as it requires consistent observation under a stereomicroscope using various identification keys. The problem is the presence of similarities in the wings for females. However, it has been shown that the presence of a clear spot under the radial cells is a typical characteristic of *C. kingi* [20]. On the other hand, according to Morag et al. [8], the presence of this spot has not been taken into account, and these species are then called atypical *C. kingi*. In fact, two morphological forms of *C. kingi* have been described [8, 21]: the Kenyan and the Senegalese forms. The two forms are separated by the presence of lightening behind the radial cells and by the spot of the second medial cell (m_2_) largely merged with its counterpart of the first medial cell (m_1_) for the Kenyan form. The Senegalese form is distinguished by the absence of lightening in the outer space of the radial cells and by a smaller spot of m_2_, which is sometimes separated from m_1_ or even absent. However, a literature review attributed atypical *C. kingi* as the corresponding *C. oxystoma* in the Japanese form described by Arnaud [21] (two spots in the cubital cell) and in Arabia by Boorman [22] (a stain in the cubital cell between rib median veins 3 and 4 [M_3 + 4_] and the edge of the wing). Cornet and Brunhes [23] reported that *C. oxystoma* is a species complex requiring taxonomic revision and that the published descriptions were not in concordance. Three different morphological features of *C. oxystoma* were found, one of which corresponded to *C. oxystoma* sensu Arnaud from Japan. Morag et al. [8] demonstrated that the other two features were present in Israel and that one of them is synonymous with the Japanese form. These authors suggest that molecular methods for analysis of cytochrome oxidase subunit I (*COI*) are useful to solve taxonomic problems associated with this group. Morphological and molecular identification techniques were used to establish the status of *C. kingi* and *C. oxystoma* present in Senegal. There are a number of PCR-based methods available to differentiate between closely related species and to supply unambiguous results. For these molecular methods, several genes have been used in large-scale *Culicoides* studies: *COI*, internal transcribed spacer 1 and 2 (*ITS1*, *ITS2*) of ribosomal DNA (*rDNA*), and the nuclear *CAD* gene [24]. The use of molecular data has renewed interest and activity in systematics [25]. The *COI* gene is the most commonly sequenced marker for *Culicoides* barcoding. However, PCR-based methods remain costly and cannot be used in routine or in large-scale epidemiological surveys. Augot et al. [26] combined a single step of PCR and restriction fragment length polymorphism (RFLP) as a tool for the identification of *Culicoides* species. This process is less time-consuming, while also enabling the processing of multiple samples simultaneously.

To the best of our knowledge, in Tunisia, no studies have been carried out combining morphological, morphometric, and molecular methods to discriminate between two closely related species in the subgenus *Remmia*: *C. oxystoma* and *C. kingi*. These species share many features, making their specific morphological identification difficult using microscopy on mounted specimens and impossible based on wing patterns using stereomicroscopy.

The aims of this study were (i) to identify *Culicoides* species trapped in different geographical regions in the district of Kairouan; (ii) to discriminate *C. oxystoma* from *C. kingi* by morphological and morphometric techniques; (iii) to develop a molecular tool combining a single step of PCR and the RFLP method to differentiate *C. oxystoma* from *C. kingi*; and (iv) to assess the phylogenetic utility to provide species identification in agreement with morphological and morphometric identifications.

## Methods

### Description of the study area

**Fig. 1:**
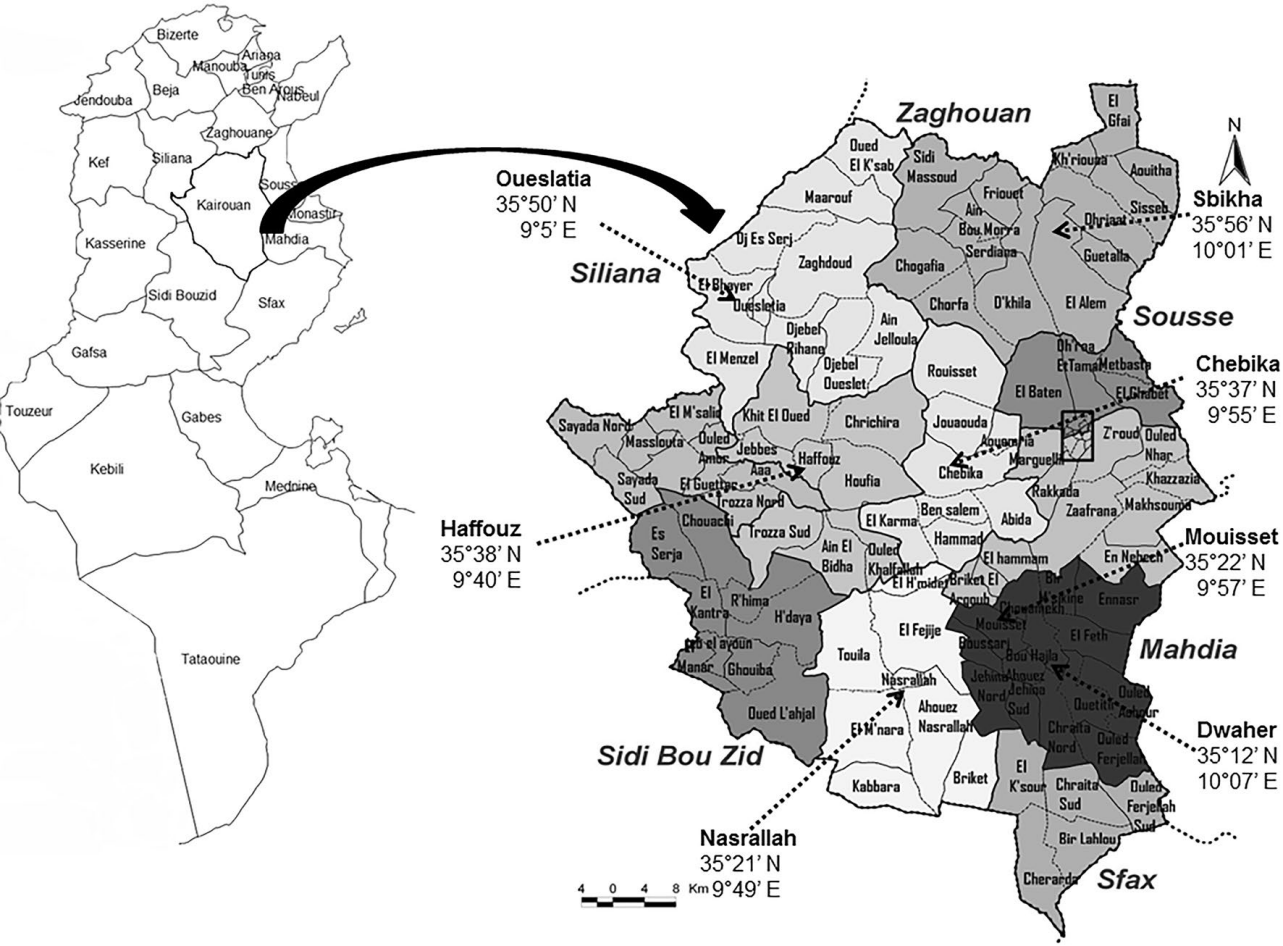
Location of study sites in Kairouan (modified according to [27]). *N* north, *E* eastern

Kairouan is located in central Tunisia and occupied an area 6712 km^2^, with a population of 570,559 (Fig. 1). This population is predominantly rural, and all collections were performed in human-inhabited biotopes with the presence of domestic animals (i.e., cattle, horses, dogs, goats, and chickens) and muddy environments that formed in proximity to livestock troughs. The region has annual rainfall of 250–400 mm. The weather of Kairouan is semiarid, with hot and dry summers and cold and wet winters.

### Field sampling

Adult midges were captured once a week from August 2014 to September 2017 in nine geographical locations within Kairouan province (Fig. 1). *Culicoides* from all sites were trapped using a Centers for Disease Control and Prevention (CDC) light trap with a collection bucket. The bucket was filled with 50 ml water to kill trapped insects. Traps were placed at sunset and collected the next day before sunrise. All specimens of *Culicoides* were stored in 70% ethanol prior to morphological and molecular analysis.

### Specimen identification and mounting

*Culicoides* midges were separated from other insects and identified according to wing characteristics using a stereomicroscope. The two species *C. kingi* and *C. oxystoma* were differentiated from each other on the basis of wing spot patterns (Additional file 1: Figure S1). The individual females were dissected on a slide using sterile dissecting needles. The head (dorsal side up), wings, and posterior abdominal segment (ventral side up) of each of these specimens were subsequently mounted on the slide under three separate coverslips using Canada balsam. The remaining thorax, legs, and anterior abdomen were stored in 75% ethanol for further molecular analysis. All identifications were performed using the Interactive Identification Key for *Culicoides* (IIKC) [28].

### Morphometric analysis

#### Morphometric measurements

Slide-mounted specimens were examined and measured using binocular microscopy. Morphometric measurements were taken from the head, wings, and genitalia of individual females. Thirteen variables (Additional file 2: Figure S2) were recorded in *Culicoides* adult females: length of the wing from arculus to tip and width of wing from the location of the second radial cell to the base of the cubital vein (Cu_1_); length of the space of the two sensilla up the eyes; length of the five flagellar segments (11 to 15) and eight basal flagellar segments (3 to 10); length of the third segment of palpus and width of the third segment of palpus; length of flagellomeres 10 and 11; length and width of the first spermatheca; length and width of the second spermatheca. Six additional variables were calculated to determine the following ratios: wing ratio (wing length/wing width); spermathecal 1 ratio (spermatheca 1 length/spermatheca 1 width); spermathecal 2 ratio (spermatheca 2 length/spermatheca 2 width); palpal ratio (length of the third palpus/length of the first and second palpi); flagella ratio (length of flagellomere 11/length of flagellomere 10 (R: 11/10); antenna segment ratio (total length of five apical segments 11–15/total length of eight basal segments 3–10).

### Statistical analysis

Here, we employed principal component analysis (PCA) to explore the correlation structure between variables and to determine which variables represented the greatest variance. PCA was carried out using Statistical Package for the Social Sciences (SPSS) version 22 (IBM Corp., Armonk, NY, USA) software. PCA calculates the correlation matrix, the principal component loading matrix, and respective eigenvalues to explain the structure of the parameters.

### Molecular analysis

#### Extraction of genomic DNA

Genomic DNA was extracted from 30 individual *Culicoides* specimens (10 *C. kingi* and 20 *C. oxystoma*) using tissue extraction kits (QIAGEN, Hilden, Germany) according to the manufacturer’s instructions. DNA samples were eluted in 100 μl of Tris EDTA (TE) buffer and stored at −20 °C. Primer sequences used for the detection of mitochondrial DNA (mtDNA) have been described previously [29]. For the cytochrome oxidase I (*COI*) gene, PCR was performed in a 50 µl volume using 8 μl of the sample extracted or control DNA (water), two units of GoTaq DNA polymerase (Promega, Madison, WI, USA), 10 μl of associated 2× buffer containing MgCl_2_, 200 μM dNTPs, and 1 μM of each primer. PCR was carried out in a GeneAmp PCR System 9700 thermal cycler under the following program [30]. Five microliters of each amplified product was visualized after electrophoresis at 100 V for 50 min with 100-bp DNA ladder molecular weight markers (Boiron, Germany) in a 1.5% agarose gel stained with 5 µl ethidium bromide. The target DNA was visualized on an ultraviolet transilluminator. The PCR product of the *COI* gene was approximately 689 bp.

#### PCR–RFLP analysis

In silico screening for a rapid PCR–RFLP discriminative method between *C. oxystoma* and *C. kingi* was first carried out using an in silico assay. Sites for restriction enzymes were predicted for *COI* sequences of *C. oxystoma* and *C. kingi* (Table 1) using Restriction Analyzer (http://www.molbiotools.com/restrictionanalyzer.html) and CLC Sequence Viewer 8.0 (www.qiagenbioinformatics.com) software. A panel of restriction enzymes was tested. One restriction enzyme, SspI, provided an original digestion pattern per species for the mtDNA marker and was selected (Additional file 3: Figure S3).

**Table 1:** In silico analysis showing the size of the mitochondrial *COI* region from five *Culicoides* species and 10 NCBI accessions, and their restriction pattern after digestion with SspI

*Culicoides* species	NCBI GenBank accession no.	PCR product (bp)	Restriction pattern (bp)	Geographical origin
*C. oxystoma*	MH135787	707	385, 233, 89	China
*C. oxystoma*	MF399786	683	385, 207, 91	Lebanon
*C. oxystoma*	MF399784	660	385, 182, 93	Lebanon
*C. kingi*	KJ729983	690	615, 75	Tunisia
*C. kingi*	KF682495	472	371, 101	Senegal
*C. enderleini*	MF399776	681	490, 161	Benin
*C. enderleini*	KJ833714	472	385, 81, 6	Senegal
*C. nevilli*	MF399696	687	385, 191, 111	Madagascar
*C. subshultzei*	KF682525	802	701, 101	South Africa
*C. subshultzei*	MH340014	564	385, 161, 18	Namibia

bp: base pairs

In vitro PCR–RFLP assays were performed in a 20 μl total volume reaction mixture containing 7 μl of PCR product (from PCR vials), 0.4 μl of SspI, and 0.8 μl of supplied buffer. PCR products were digested for 1 h at 37 °C. The digested samples were separated by electrophoresis on a 2% agarose gel to produce DNA fragments and were sized by comparison with a 100-bp marker ladder (Boiron, Germany).

#### DNA sequencing and phylogenetic analysis

To confirm the results of the PCR–RFLP analysis, direct sequencing of the *COI* gene amplicons was performed using the same set of primers that were used in the PCR assay (Eurofins MWG Operon, Munich, Germany). The obtained sequences were edited using Chromas software version 2.33 (http://ww.technelysium.com. au/chromas.html) and identified by comparison with sequences available in GenBank using the Basic Local Alignment Search Tool (BLAST) (www.ncbi.nlm.nih.gov/blast/). Species assignment was considered complete when a match of 98% or greater was found between our sequences and those in GenBank. DNA sequence-based analyses were performed using the maximum likelihood (ML) method (Tamura-Nei) with MEGA 5 software [31]. Genetic distances were computed using the Kimura 2-parameter (K2P) method. The tree topology was supported by 1000 bootstrap replicates to determine node reliability. *COI* sequences in this study were compared with sequences from the same species in GenBank (MF399736, MF399686, MF399705, MF399698, KJ729983, MF399780, MF399688, MF399776, MF399699, MF399697).

## Results

### Morphological identification

**Fig. 2:**
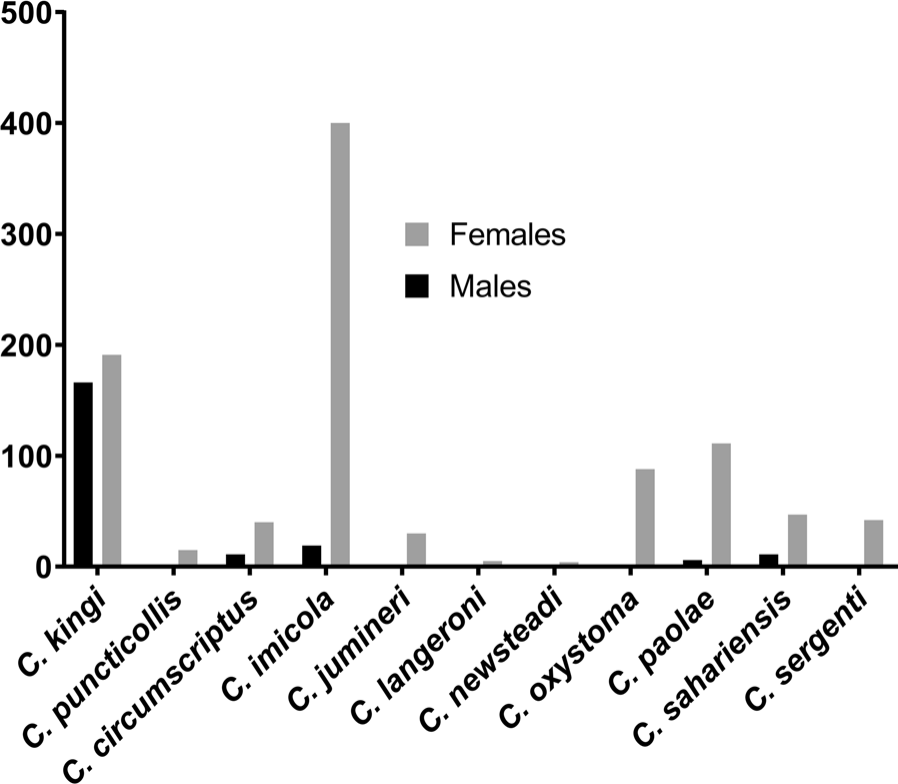
*Culicoides* species collected in the district of Kairouan according to sex

During the study period, 1190 biting midges were collected in the district of Kairouan (Fig. 2). Of these specimens, 82% and 18% females and males were identified, respectively. Morphological identification revealed the presence of 11 species: *C. imicola*, *C. kingi*, *C. paolae*, *C. oxystoma*, *C. sahariensis*, *C. circumscriptus*, *C. sergenti*, *C. jumineri*, *C. puncticollis*, *C. langeroni*, and *C. newsteadi*.

*Culicoides imicola* and *C. kingi* were the most abundant species, constituting 35.21% (*n* = 419) and 30% (*n* = 357), respectively (Fig. 2). Two species belonging to the *Schultzei* group were identified that were ascribable to *C. kingi* (190 ♀, 167♂) and *C. oxystoma* (88 ♀, 01 ♂). It should be noted that *C. oxystoma* is reported here for the first time in Tunisia. This species was collected from the district of Sbikha (Kairouan) (Fig. 1). However, *C. kingi* was more abundant and was sampled at the four collection sites. The wing structure was generally unique for each species, although slight variations in wing patterns were observed between the two species (Additional file 1: Figure S1). *Culicoides kingi* from Tunisia presented two pale round spots after the second radial cell (r_2_), one pale spot in the first medial cell (m_1_ of the wing crossing the median vein (M_2_), two separate pale spots in m_4_, two pale spots in anal cells (An), and one pale spot in m_1_ and m_2_ (Additional file 1: Figure S1). *Culicoides oxystoma* had pale spots under radial cells, pale spots in m_1_ that did not cross M_2_, another pale spot between Cu_1_) and the edge of the wing, and two pale spots merged in m_4_ (Additional file 1: Figure S1).

### Morphometric analysis

**Fig. 3:**
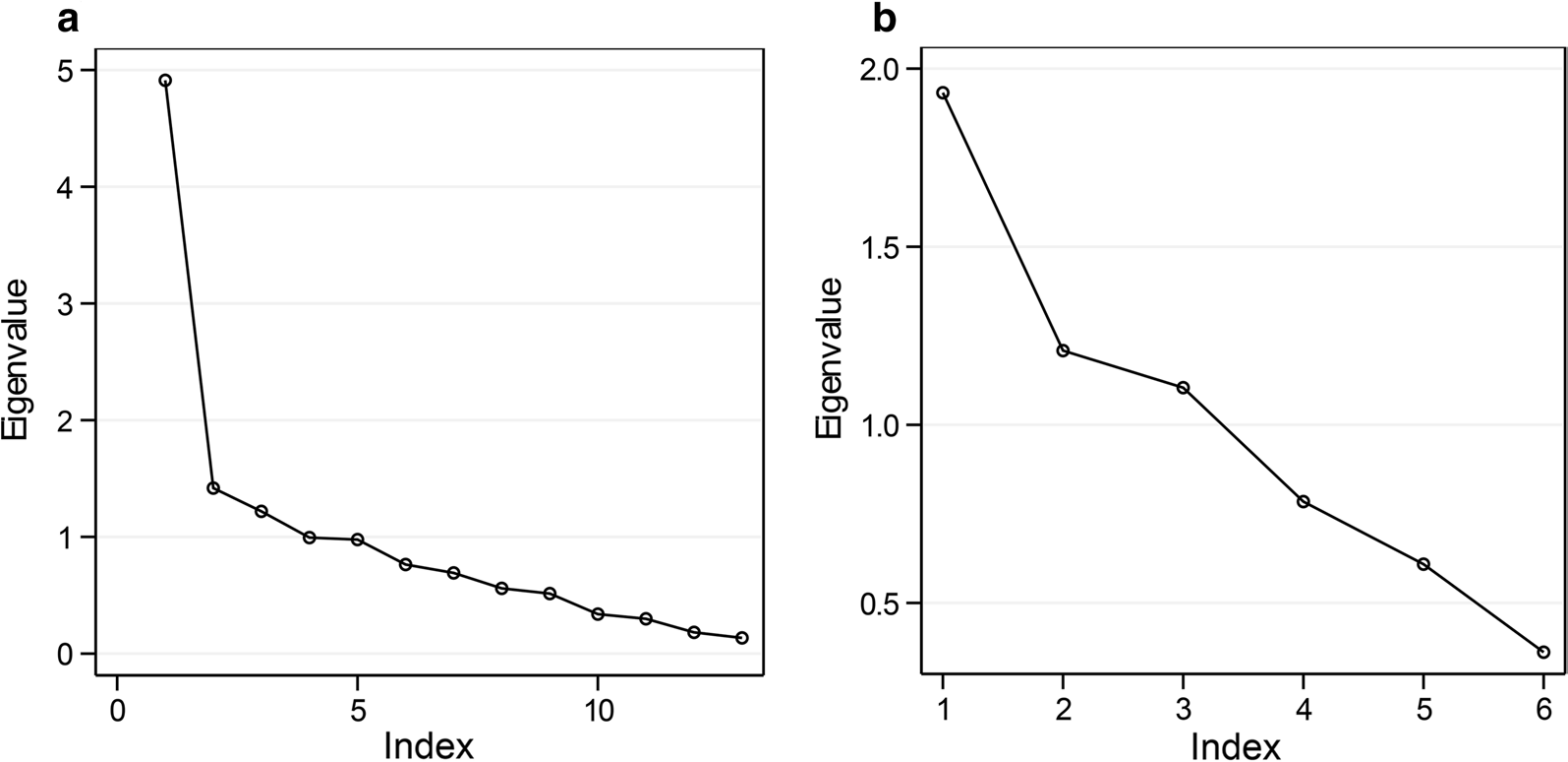
**a** A scree plot highlighting the relationship between the eigenvalues and the number of axes in PCA of 13 morphometric measurements of *C. kingi* and *C. oxystoma*; **b** six ratios derived from morphometric measurements of those individuals

Morphometric differences in the measurement data were studied via PCA. Kaiser’s [32] stopping rule states that only the number of axes with eigenvalues over 1.00 should be considered in the analysis. From the 13 morphometric measurements, five principal components (axes) had an eigenvalue greater than 1.00 (Table  2), and when combined, these factors accounted for 72.24% of the total variance. A scree plot (Fig. 3) suggested inclusion of only the two first axes PC1 and PC2.

**Table 2:** The eigenvalues, percent variance, and cumulative variance of the axes from PCA of 13 morphometric measurements of *C. kingi* and *C. oxystoma*

Axis (principal component)	Initial eigenvalues		
	Total	% of variance	Cumulative %
1	4.624	35.566	35.566
2	1.447	11.130	46.696
3	1.267	9.744	56.440
4	1.037	7.976	64.416
5	1.018	7.830	72.246
6	0.779	5.995	78.241
7	0.730	5.616	83.857
8	0.570	4.388	88.246
9	0.515	3.962	92.207
10	0.346	2.658	94.865
11	0.319	2.453	97.318
12	0.199	1.529	98.847
13	0.150	1.153	100.000

The first axis (PC1) was positively correlated with the length of eight basal segments (loadings ≥ 0.8) and with the lengths and widths of the wing (loadings ≥ 0.65) and correlated with the third palpal segment and the length of flagellomere 11 (loadings ≥ 0.65). The second axis (PC2) was positively correlated with the length of spermathecae 1 and 2, as well as the width of the third segment palpus (loadings ≥ 0.4). Additionally, it correlated with the length of five apical segments and with the length of flagellomere 10 (loadings ≥ 0.5) (Table  3).

**Table 3:** Characterization of *C. kingi* and *C. oxystoma* using the loadings of PCA on 13 morphometric parameters

Parameter	Principal component				
	PC1	PC2	PC3	PC4	PC5
Length of the 8 basal segments	0.904	0.085	0.003	0.144	0.067
Wing length	0.720	0.356	0.297	0.012	0.368
Wing width	0.653	0.385	0.409	0.063	0.244
Length of flagellomere 11	0.613	0.372	0.314	−0.101	0.023
Width of the third palpus	0.540	0.418	0.093	0.366	0.107
Spermatheca 1 length	0.045	0.814	0.061	−0.158	0.205
Length of the flagellar segment	0.240	0.718	0.211	0.047	−0.040
Length of flagellomere 10	0.267	0.512	−0.107	0.362	0.311
Spermatheca 2 width	0.107	0.001	0.885	−0.016	0.183
Spermatheca 2 length	0.259	0.439	0.633	0.176	−0.183
Length third palpus	0.101	−0.044	0.001	−0.842	0.097
Length of the space of the 2 sensilla up the eye	−0.035	−0.314	0.306	0.534	0.360
Spermatheca 1 width	0.101	0.161	0.084	−0.010	0.872

Rotation method: varimax with Kaiser normalization

PCA was also applied to the six morphometric ratios to augur differences in shape between *C. kingi* and *C. oxystoma*. Kaiser’s stopping rule suggested the inclusion of the first three axes (Table 4), while the screen test (Fig. 3) suggested inclusion of first two axes. Indeed, four of the six axes accounted for similar amounts of variance (6–18%) (Table 5). PC1 was correlated with the palpal and flagella ratios, while PC2 was negatively correlated with the segment ratio and positively correlated with the spermatheca ratio (Table 4).

**Table 4:** Characterization of *C. kingi* and *C. oxystoma* using the loadings of PCA on six ratios derived from morphometric parameters

	Principal component		
Parameter	PC1	PC2	PC3
Palpal ratio	0.898	−0.130	0.010
Flagella ratio	0.828	0.316	0.098
Antenna segment ratio	0.065	−0.827	0.076
Spermatheca 2 ratio	0.204	0.783	0.189
Wing ratio	−0.053	−0.127	0.829
Spermatheca 1 ratio	0.154	0.240	0.680

**Table 5:** The eigenvalues, percent variance, and cumulative variance of the axes from PCA of six ratios derived from morphometric measurements of *C. kingi* and *C. oxystoma*

Axis (principal component)	Initial eigenvalues		
	Total	% of variance	Cumulative %
1	1.917	31.947	31.947
2	1.229	20.480	52.427
3	1.107	18.448	70.875
4	0.789	13.156	84.031
5	0.590	9.829	93.860
6	0.368	6.140	100.000

### Molecular analyses

PCR amplification of the *COI* region of the mtDNA from the midges identified morphologically as *C. kingi* and *C. oxystoma* (*n* = 30) exhibited uniformity in band size (689 bp).

#### Species-diagnostic restriction enzyme sites

In silico PCR–RFLP simulation using different restriction enzymes demonstrated that SspI was suitable for discrimination between the closely related species *C. oxystoma* and *C. kingi*. The restriction fragments obtained from each DNA reference sample (Table 1) agreed with the predicted profiles for the *COI* sequence by in silico analysis (Additional file 3: Figure S3).

Digestion of the *COI* PCR products with SspI demonstrated that the patterns of the two species were different. For *C. kingi* (KJ729983.1), two restriction fragments were predicted (615 and 74 bp), but in agarose gel only one band (615 bp) could be seen, probably because of co-migration of the 74-bp band (Additional file 3: Figure S3). For *C. oxystoma*, three very close bands were visible in agarose gel (385, 210, 994 bp) (Additional file 3: Figure S3).

#### DNA sequencing and phylogenetic analysis

**Fig. 4:**
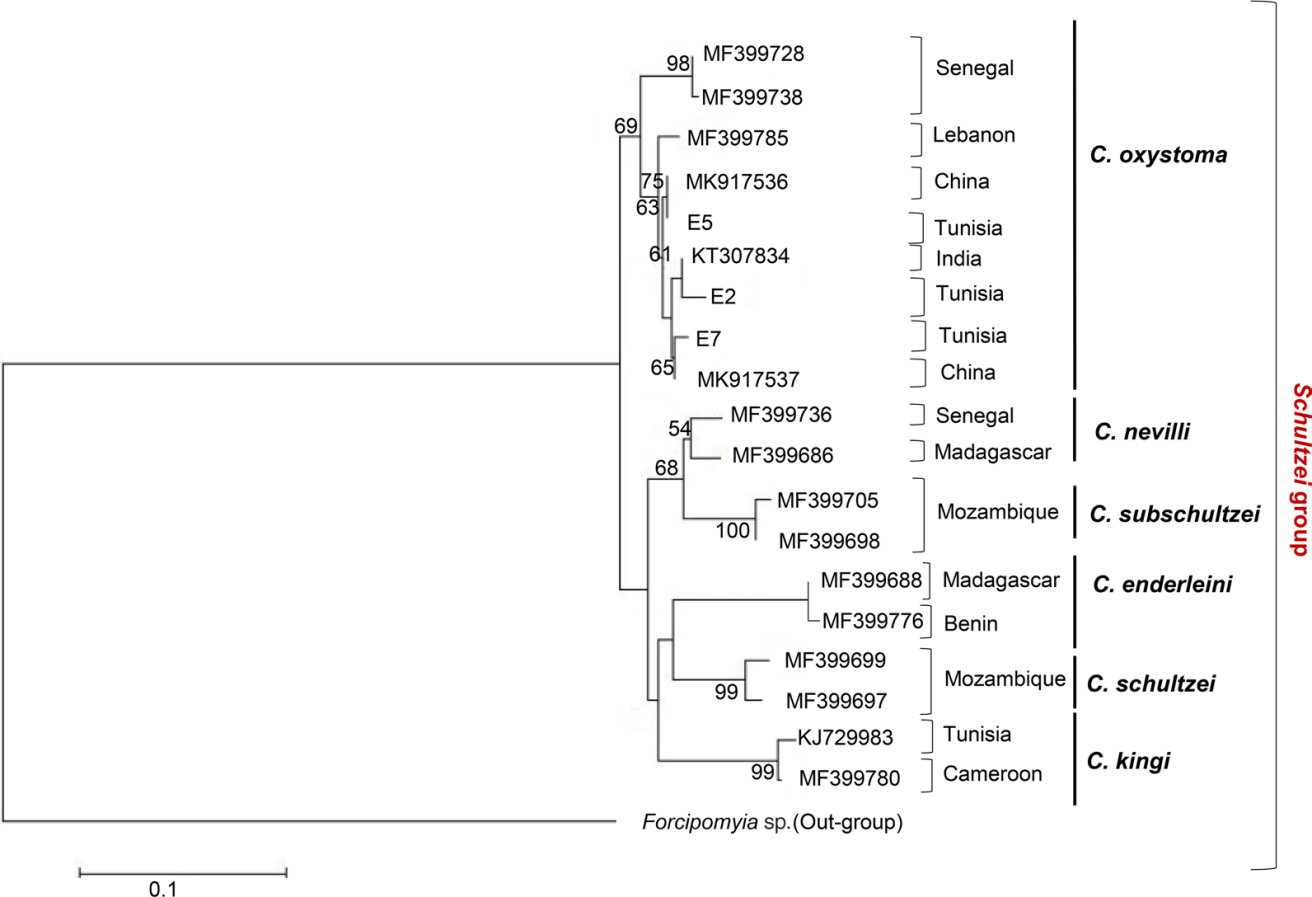
Maximum likelihood tree based on *COI* nucleotide sequences obtained in this study. The tree show phylogenetic analyses of three individual midges morphologically identified as *C. oxystoma* together with the most closely related published sequences and their GenBank accession numbers. Bootstrap values are shown on the branches

The obtained DNA sequences were compared with those deposited in the GenBank database. Phylogenetic analysis was performed to confirm the genetic relationship between species. The topology of the phylogenetic tree showed a clear subdivision in seven distinct and well-supported phylogenetic lineages corresponding to six species of the *Schultzei* group (*C. oxystoma*, *C. kingi*, *C. nevilli*, *C. subschultzei*, *C. enderleini*, and *C. schultzei*), and *Forcipomyia* sp. Specimens of *C. oxystoma* formed two separate clades, one including specimens from India (KT307834) and China (MK917536; MK917537) and the other including specimens from Senegal (MF399728 and MF399738). Tunisian specimens (E2, E5, and E7) identified as *C. oxystoma* and representing morphological variations (Additional file 1: Figure S1) were grouped in the same clade. One clade clustered the specimens identified as *C. kingi*, including those collected in Tunisia (KJ729983) and Cameroon (MF399780) (Fig. 4).

## Discussion

To the best of our knowledge, this is the first study on the presence of *C. oxystoma* in Tunisia. However, this is also the first morphological and molecular investigation aiming to discriminate *C. oxystoma* from *C. kingi*.

*Culicoides imicola* was the dominant species of biting midges recorded during this study, representing 35.21% of the total *Culicoides* captured by CDC light traps. This result is consistent with previous findings in Tunisia [33–36], where *C. imicola* was typically the most dominant species, exceeding 80% of the total species composition and abundance at sites where BTV transmission was intense. The second most dominant species collected in this study comprised midges of the *Schultzei* group, *C. kingi*. This species represented 30% of the total *Culicoides* captured. Other species were collected but in minor numbers. It is important to note that the main weakness with this low proportion (e.g., *C. sahariensis* and others) is the inability for subsampling [37]. Furthermore, as traps were only operated from 1 h before dusk until 1 h after sunrise, *Culicoides* spp. that are active during daylight hours were likely underrepresented. However, Sanders et al. [38] found that estimation based on subsampling provided a good estimation of the total *Culicoides* captured, and it has been previously demonstrated that most *Culicoides* spp. are crepuscular [3].

The identification of *C. oxystoma* in Tunisia (Kairouan) is of great importance, as this species is known to transmit many important bovine arboviruses, including Akabane virus [7, 39]. Additionally, it is a suspected vector of EHDV in Israel [8]. It would therefore be interesting to determine whether this important potential vector species plays a role in the transmission of *Culicoides*-borne diseases in Tunisia and North Africa. This species shares many morphological characteristics with *C. kingi*, making morphological identification very difficult. Many previous studies have tried to separate closely related *Culicoides* species such as *C. obsoletus* and *C. scoticus*, and a number of studies [1, 39, 40] have been conducted to assess the reliability of traditional morphological or morphometric identification techniques to differentiate between the two species. Augot et al. [1] suggested that *C. obsoletus* and *C. scoticus* can be identified using multivariate analyses based on the length and width of spermatheca 1, the length of spermatheca 2, and the width between the chitinous plates. Foxi et al. [41] identified females in the *Obsoletus* group under a stereomicroscope by combining two characteristics: the shape of the third segment of the maxillary palp, and the number and location of hairs on the first abdominal tergite. It should be noted that previous studies have indicated that measurements of the total length of the five apical segments and eight basal segments of the antenna, which produce an antenna ratio, can significantly differentiate *C. obsoletus* and *C. scoticus.* Furthermore, a study by Kluiters et al. [42] highlighted that abdominal measurements, such as larger and smaller spermatheca lengths and widths, can be used to differentiate *C. obsoletus* from *C. scoticus.*

In the current study, the diagnostic value of 13 morphometric variables in females of *C. kingi* and *C. oxystoma* was used to verify statistical identification. The results suggest that abdominal measurements (length of eight basal segments and wing lengths and widths) could be used to reliably separate these two species, despite the small numbers of samples and individuals used, as *C. kingi* exhibits smaller measurements than those from *C. oxystoma*. Our research demonstrates that abdominal size combined with the form of the wing spot is a suitable characteristic for separating the two species.

We developed a PCR–RFLP-based tool to discriminate between the species *C. kingi* and *C. oxystoma*. Interestingly, it was found that this approach was able to produce different profiles using the SspI restriction enzyme and could therefore eliminate the difficulties present in systematic identification. Each of the two species can be discriminated by visualizing agarose gel in an ultraviolet transilluminator, as each species produces a unique pattern after restriction digestion. Furthermore, the same results were obtained with in silico digestion, demonstrating that *C. oxystoma* was distinguishable from *C. kingi.* Compared to classical molecular techniques, this technique is rarely used for the identification of *Culicoides* species [37, 43]; our PCR–RFLP method produced satisfying results and could be a convenient tool to separate the two closely related species.

There has been much discussion of the taxonomic status of *C. oxystoma* as a member of the *Schultzei* group, but it remains unclear [21, 44]. Here, we found that the morphologically and molecularly identified *C. oxystoma* from the present study was closely related to *C. oxystoma* from India, China, Israel, and Lebanon, and was separated from those of Senegal. Our results were consistent with those previously reported [8, 45, 46]. The maximum likelihood tree also revealed that *C. oxystoma* is discriminated from *C. kingi*. This result is consistent with morphometric analyses showing a high degree of divergence between the two species.

Furthermore, it was suggested that *C. oxystoma* is a complex of sibling species [21, 47], owing to the high level of intraspecific divergence observed in *C. oxystoma* based on the *COI* sequences [46, 48]. Further studies on a large number of specimens are needed to study the taxonomic status of *C. oxystoma* species in Tunisia.

## Conclusions

In this study, we highlighted that *C. oxystoma* could be differentiated based on wing form and abdominal measurements. Accordingly, a PCR–RFLP assay was developed to discriminate between these two closely related species. This technique is cost-effective and rapid and does not require any sophisticated equipment. Characterization of cryptic species could be conducted using PCR–RFLP, and its utilization could be more common in the future. Finally, combining morphological and molecular identification of *Culicoides* specimens is very important for a better understanding of the systematics of *C. oxystoma*, which is a vector of disease.

## Supplementary Information

**Additional file 1: Figure S1.** Wing structure of *Culicoides* species. **a**. Wings of *C. kingi* (**b1**) and *C. oxystoma* (**b2**), demonstrating the difficulty in differentiating between species of the *Schultzei* group. *Arc* arculus, *Trans* transverse, *r*^1^, *r*^2^ first and second radial cells, *r*^5^ radial cell, *M1, M2* first and second median veins, *Cu*^1^, *Cu*^2^ first and second cubital veins, *An* anal cell; *m*^1^, *m*^2^ first and second medial cell.

**Additional file 2: Figure S2.** Morphometric measurements of *Culicoides* species. **a** Wing (length wing [a1], width wing [a2]). **b** Head (length of the space of the two sensilla up the eyes [b1]; length of the five flagellar segments [b2] and eight basal flagellar segments [b3]; length of the third palpus [b4] and width of the third segment of the palp [b5]; length of flagellomeres 10 [b6] and 11 [b7]). **c** Spermathecae (length [c1] and width of the first spermatheca [c2]; length [c3] and width of the second spermatheca [c4]). Different lowercase letters indicate the measurements taken on different parts of the *Culicoides* body.

**Additional file 3: Figure S3.** PCR products (lanes 1–4) of the *Culicoides* species (**a**). Lane C: negative control (no DNA); lane MW: molecular marker 1 Kb plus DNA ladder (Invitrogen™). **b** Restriction results revealing complete coincidence with the in silico analysis. **c** In silico analysis of restriction profiles using SspI for the same species (S1–S4, *C. oxystoma*; S5, *C. kingi*, accession number: KJ729983). *M* molecular marker 1 Kb.

## Data Availability

Not applicable.

## Abbreviations

PCR: Polymerase chain reaction
RFLP: Restriction fragment length polymorphism
BTV: Bluetongue virus
AHS: African Horse Sickness
EHDV: Epizootic hemorrhagic disease virus
SBV: Schmallenberg virus
COI: Cytochrome oxidase subunit I
ITS1: Internal transcribed spacer 1
ITS2: Internal transcribed spacer 2
rDNA: Ribosomal DNA
CDC: Centers for Disease Control and Prevention
IIKC: Interactive Identification Key for *Culicoides*
Cu1: Cubital 1
PCA: Principal component analysis
SPSS: Statistical Package for the Social Sciences
DNA: Deoxyribonucleic acid
EDTA: Ethylenediamine tetraacetic acid
TE: Tris ethylenediamine tetraacetic acid
MgCl_2_: Magnesium chloride 2
dNTP: Deoxyribonucleotide triphosphate
BLAST: Basic Local Alignment Search Tool
ML: Maximum Likelihood
K2P: Kimura 2-parameter
r_2_: Second radial cell
m_1_: First medial cell
M_2_: Median vein
An: Anal cell
PC1: Principal component 1
PC2: Principal component 2
