# Supplementary figures and images for: Morphological and molecular differentiation between Culicoides oxystoma and Culicoides kingi (Diptera: Ceratopogonidae) in Tunisia
Source: Parasit Vectors. 2021 Dec 18;14:607. doi: 10.1186/s13071-021-05084-8 (PMC8684274; doi:10.1186/s13071-021-05084-8)

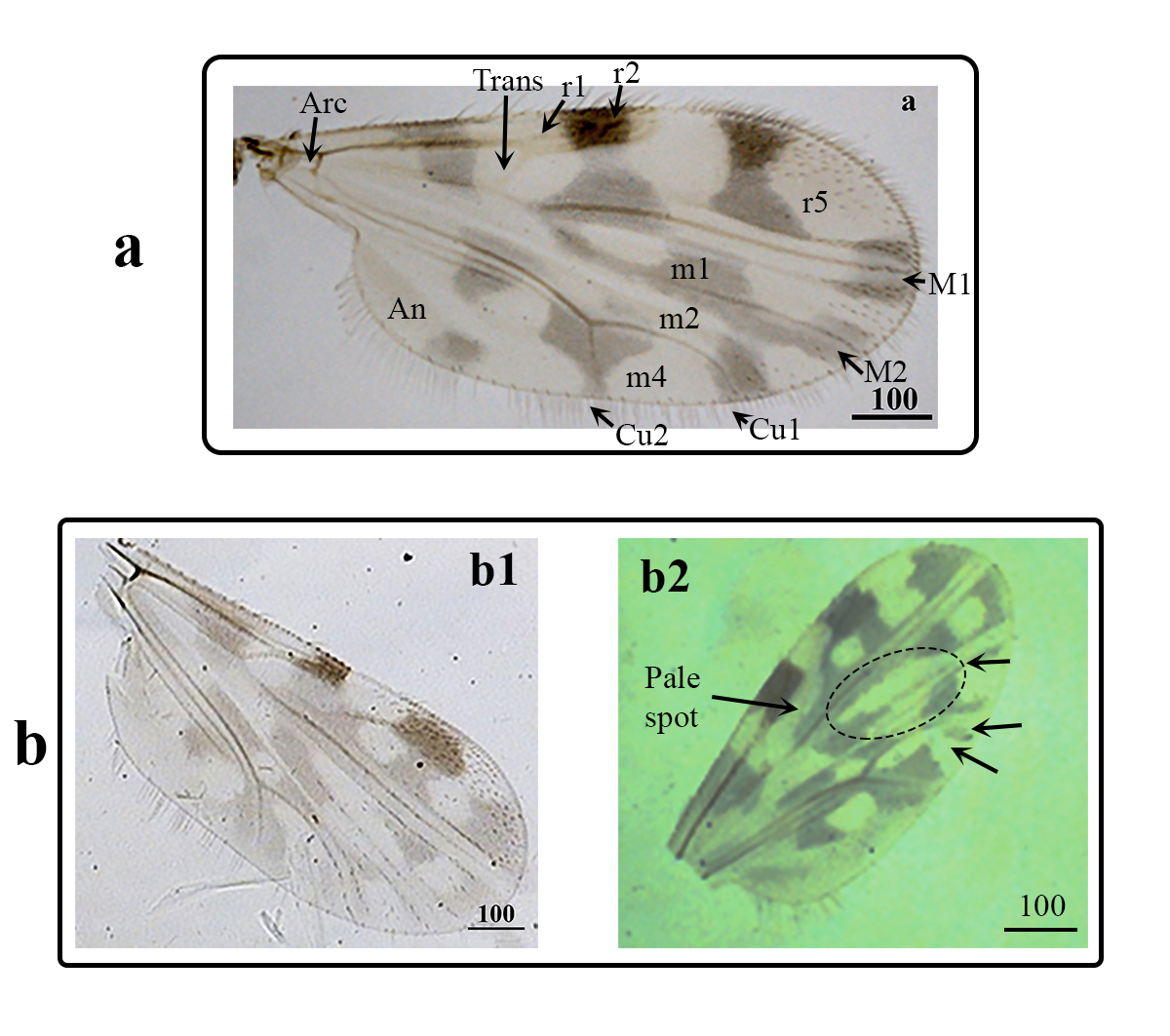

Supplement: Supplementary file 1 — Additional file 1: Figure S1. Wing structure of Culicoides species. a. Wings of C. kingi (b1) and C. oxystoma (b2), demonstrating the difficulty in differentiating between species of the Schultzei group. Arc arculus, Trans transverse, r1, r2 first and second radial cells, r5 radial cell, M1, M2 first and second median veins, Cu1, Cu2 first and second cubital veins, An anal cell; m1, m2 first and second medial cell. [file 13071_2021_5084_MOESM1_ESM.jpg]

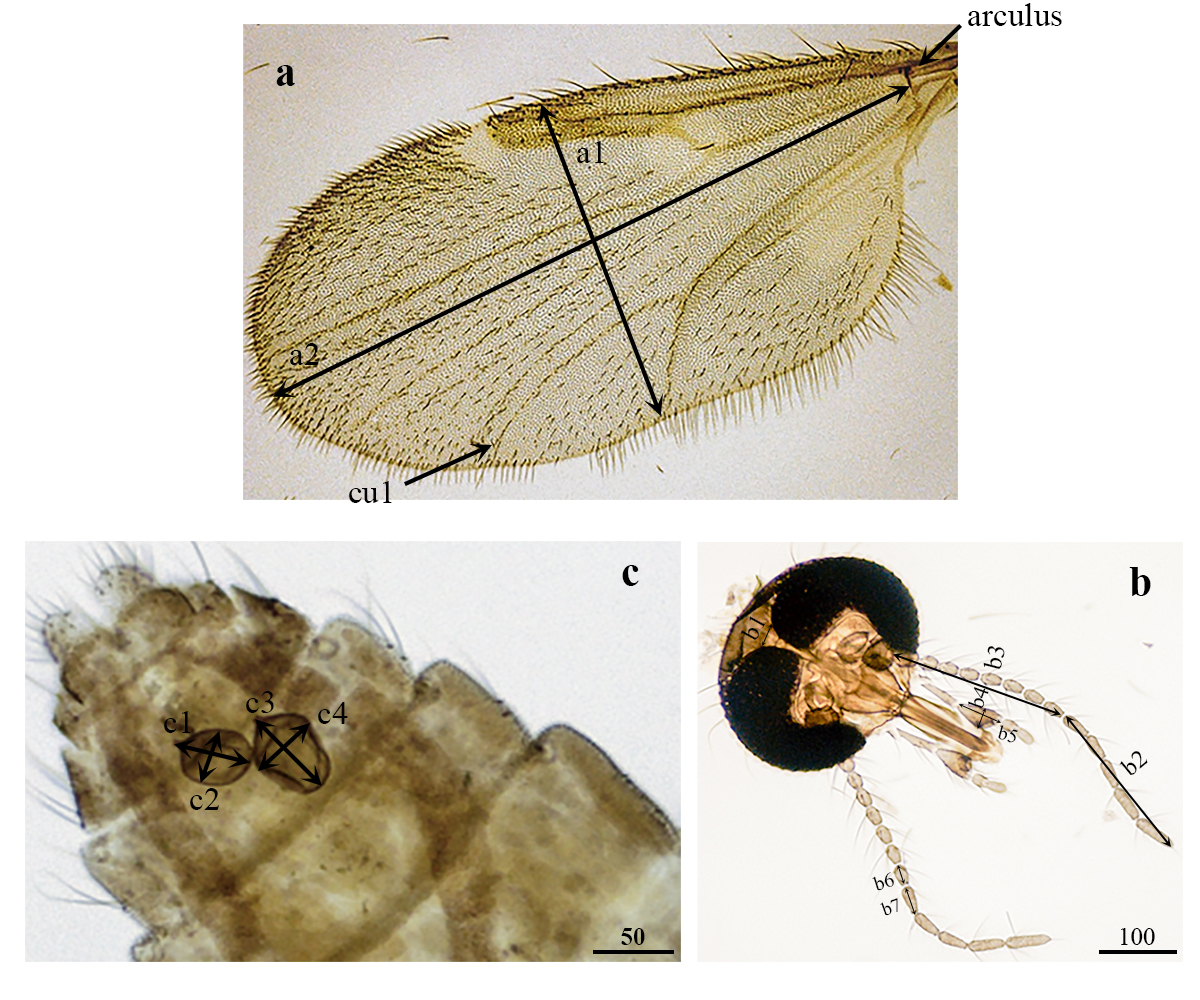

Supplement: Supplementary file 2 — Additional file 2: Figure S2. Morphometric measurements of Culicoides species. a Wing (length wing [a1], width wing [a2]). b Head (length of the space of the two sensilla up the eyes [b1]; length of the five flagellar segments [b2] and eight basal flagellar segments [b3]; length of the third palpus [b4] and width of the third segment of the palp [b5]; length of flagellomeres 10 [b6] and 11 [b7]). c Spermathecae (length [c1] and width of the first spermatheca [c2]; length [c3] and width of the second spermatheca [c4]). Different lowercase letters indicate the measurements taken on different parts of the Culicoides body. [file 13071_2021_5084_MOESM2_ESM.jpg]

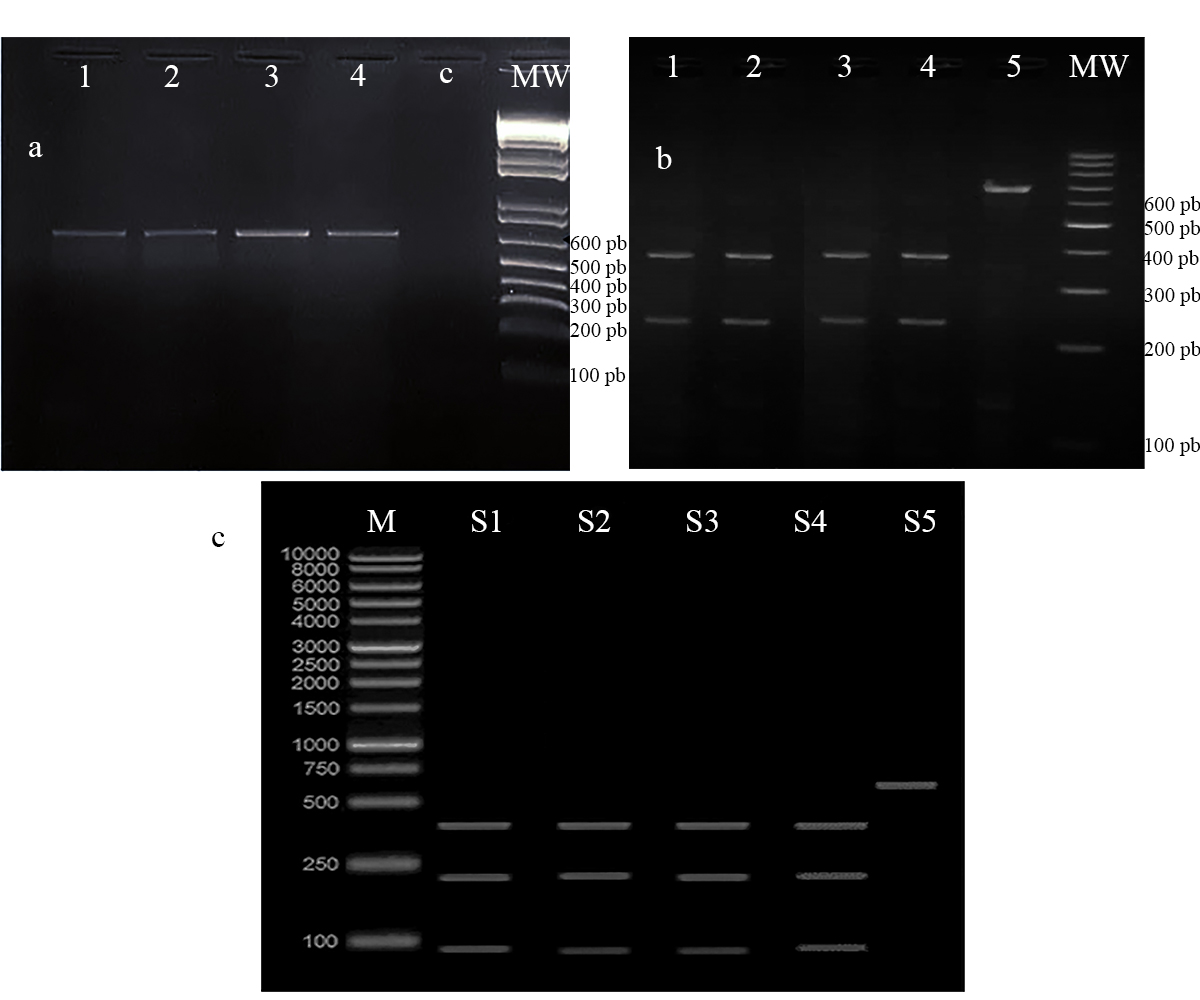

Supplement: Supplementary file 3 — Additional file 3: Figure S3. PCR products (lanes 1–4) of the Culicoides species (a). Lane C: negative control (no DNA); lane MW: molecular marker 1 Kb plus DNA ladder (Invitrogen™). b Restriction results revealing complete coincidence with the in silico analysis. c In silico analysis of restriction profiles using SspI for the same species (S1–S4, C. oxystoma; S5, C. kingi, accession number: KJ729983). M molecular marker 1 Kb. [file 13071_2021_5084_MOESM3_ESM.jpg]
